# A Potent Tartrate Resistant Acid Phosphatase Inhibitor to Study the Function of TRAP in Alveolar Macrophages

**DOI:** 10.1038/s41598-017-12623-w

**Published:** 2017-10-03

**Authors:** Carian E. Boorsma, T. Anienke van der Veen, Kurnia S. S. Putri, Andreia de Almeida, Christina Draijer, Thais Mauad, Gyorgy Fejer, Corry-Anke Brandsma, Maarten van den Berge, Yohan Bossé, Don Sin, Ke Hao, Anja Reithmeier, Göran Andersson, Peter Olinga, Wim Timens, Angela Casini, Barbro N. Melgert

**Affiliations:** 1 University of Groningen, Department of Pharmacokinetics, Toxicology and Targeting, Groningen Research Institute for Pharmacy, Groningen, The Netherlands; 2 University of Groningen, Department of Pharmaceutical Technology and Biopharmacy, Groningen Research Institute for Pharmacy, Groningen, The Netherlands; 3 School of Chemistry, Cardiff University, Cardiff, United Kingdom; 4 São Paulo University, Department of Pathology, São Paulo, Brazil; 5 University of Plymouth, School of Biomedical and Healthcare Sciences, Peninsula Schools of Medicine and Dentistry, Plymouth, United Kingdom; 6 University of Groningen, University Medical Center Groningen, Department of Pathology, Groningen, The Netherlands; 7 University of Groningen, University Medical Center Groningen, Department of Pulmonology, Groningen, The Netherlands; 8 University of Groningen, University Medical Center Groningen, GRIAC Research Institute, Groningen, The Netherlands; 9 Laval University, Institut Universitaire de Cardiologie et de Pneumologie de Québec, Department of Molecular Medicine, Québec, Canada; 10 University of British Columbia, James Hogg Research Center, Providence Heart+Lung Institute, St. Paul’s Hospital, Vancouver, British Columbia Canada; 11 University of British Columbia, Respiratory Division, Department of Medicine, Vancouver, British Columbia Canada; 12 Merck Research Laboratories, Boston, Massachusetts United States of America; 13 Karolinska Institute, Department of Laboratory Medicine (LABMED), H5, Division of Pathology, F46, Karolinska University hospital, Huddinge, Stockholm Sweden

## Abstract

The enzyme tartrate resistant acid phosphatase (TRAP, two isoforms 5a and 5b) is highly expressed in alveolar macrophages, but its function there is unclear and potent selective inhibitors of TRAP are required to assess functional aspects of the protein. We found higher TRAP activity/expression in lungs of patients with chronic obstructive pulmonary disease (COPD) and asthma compared to controls and more TRAP activity in lungs of mice with experimental COPD or asthma. Stimuli related to asthma and/or COPD were tested for their capacity to induce TRAP. Receptor activator of NF-κb ligand (RANKL) and Xanthine/Xanthine Oxidase induced TRAP mRNA expression in mouse macrophages, but only RANKL also induced TRAP activity in mouse lung slices. Several Au(III) coordination compounds were tested for their ability to inhibit TRAP activity and [Au(4,4′-dimethoxy-2,2′-bipyridine)Cl_2_][PF_6_] (AubipyOMe) was found to be the most potent inhibitor of TRAP5a and 5b activity reported to date (IC50 1.3 and 1.8 μM respectively). AubipyOMe also inhibited TRAP activity in murine macrophage and human lung tissue extracts. In a functional assay with physiological TRAP substrate osteopontin, AubipyOMe inhibited mouse macrophage migration over osteopontin-coated membranes. In conclusion, higher TRAP expression/activity are associated with COPD and asthma and TRAP is involved in regulating macrophage migration.

## Introduction

Tartrate resistant acid phosphatase (TRAP) is a metalloenzyme and a member of the purple acid phosphatases, containing a binuclear iron (Fe^3+^/Fe^2+^) center that facilitates the hydrolysis of phosphate esters and the generation of reactive oxygen species (ROS)^1–5^. It is highly expressed in osteoclasts and alveolar macrophages and lower expression can be found in activated macrophages and dendritic cells^6–9^.

TRAP exists in two isoforms: the 5a isoform is a monomer, while the 5b isoform is a dimer derived from 5a by proteolytic cleavage of a repressive loop domain and is the enzymatically more active form^1,10–12^. Alveolar macrophages have especially high expression of TRAP5a while osteoclasts express high levels of TRAP5b^6,7,13^. The function of TRAP5b in bone has been studied in relation to bone remodeling extensively, in which TRAP activity was found to mediate osteoclast migration^2,14,15^. Osteoclasts are attached to bone matrix through an osteopontin - integrin alphav-beta3 (α_v_β_3_) bond. Migration of osteoclasts is promoted when this bond is disconnected by TRAP-dependent dephosphorylation of osteopontin.

The role of TRAP5a in alveolar macrophages has not been clarified yet but it has been postulated to play a role in bacterial killing by its ability to generate ROS^16^. In addition, little is known about the regulation of TRAP expression in alveolar macrophages. Two studies investigated the expression of TRAP in lung tissue and another specifically measured TRAP expression in alveolar macrophages and all found higher expression in smokers^17–19^. Therefore, we investigated whether its expression and/or activity are also altered in patients with chronic obstructive pulmonary disease (COPD) and other obstructive respiratory diseases like asthma and which disease-specific conditions can change TRAP expression/activity.

**Figure 1:**
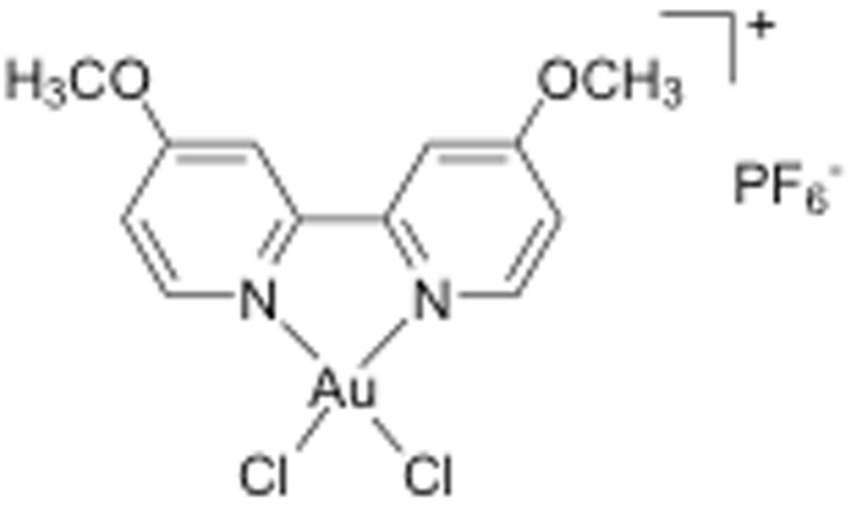
Chemical structure of the Au(III) compound [Au(4,4′-dimethoxy-2,2′-bipyridine)Cl_2_][PF_6_] (AubipyOMe).

Exploring the function of TRAP activity in the lung has been hampered by the availability of only few inhibitors that either have low potency, low stability or are toxic^15,20–24^. Hayman *et al*. demonstrated potent inhibitory effects of sodium tetrachloroaurate (NaAuCl_4_) on TRAP activity^20^. However, this Au(III) complex is a reactive compound prone to reduction in biological environment and has unspecific protein binding, which may interfere with many different cellular pathways. In recent years, gold-based compounds of different families have been shown to possess ideal enzyme/protein inhibition properties, which allow them to be designed and exploited as chemical probes to study protein functions in biological systems and to possibly be developed as therapeutic agents^25–28^. Thus, a series of gold coordination complexes with N-donor ligands, conferring stability to Au(III) ions, were screened for TRAP inhibition *in vitro*. Among the newly tested gold complexes, the compound [Au(4,4′-dimethoxy-2,2′-bipyridine)Cl_2_][PF_6_] (AubipyOMe, Fig. 1) was found to be the most potent inhibitor of TRAP activity described to date.

We subsequently used AubipyOMe to study the function of TRAP in macrophages. Our starting hypothesis was that TRAP activity is also involved in regulation of osteopontin-dependent macrophage migration, similar to osteoclasts in bone. Osteopontin is expressed on the luminal side of epithelial cells and alveolar macrophages are present in the lumen of airways and alveoli^29^. Alveolar macrophages also express α_v_β_3_ integrins and we hypothesized that they may also need TRAP to migrate^30^. Therefore, we used AubipyOMe to investigate functional aspects of TRAP activity in macrophages, such as cell migration.

## Results

### TRAP expression is higher in smokers and in patients with COPD

**Figure 2:**
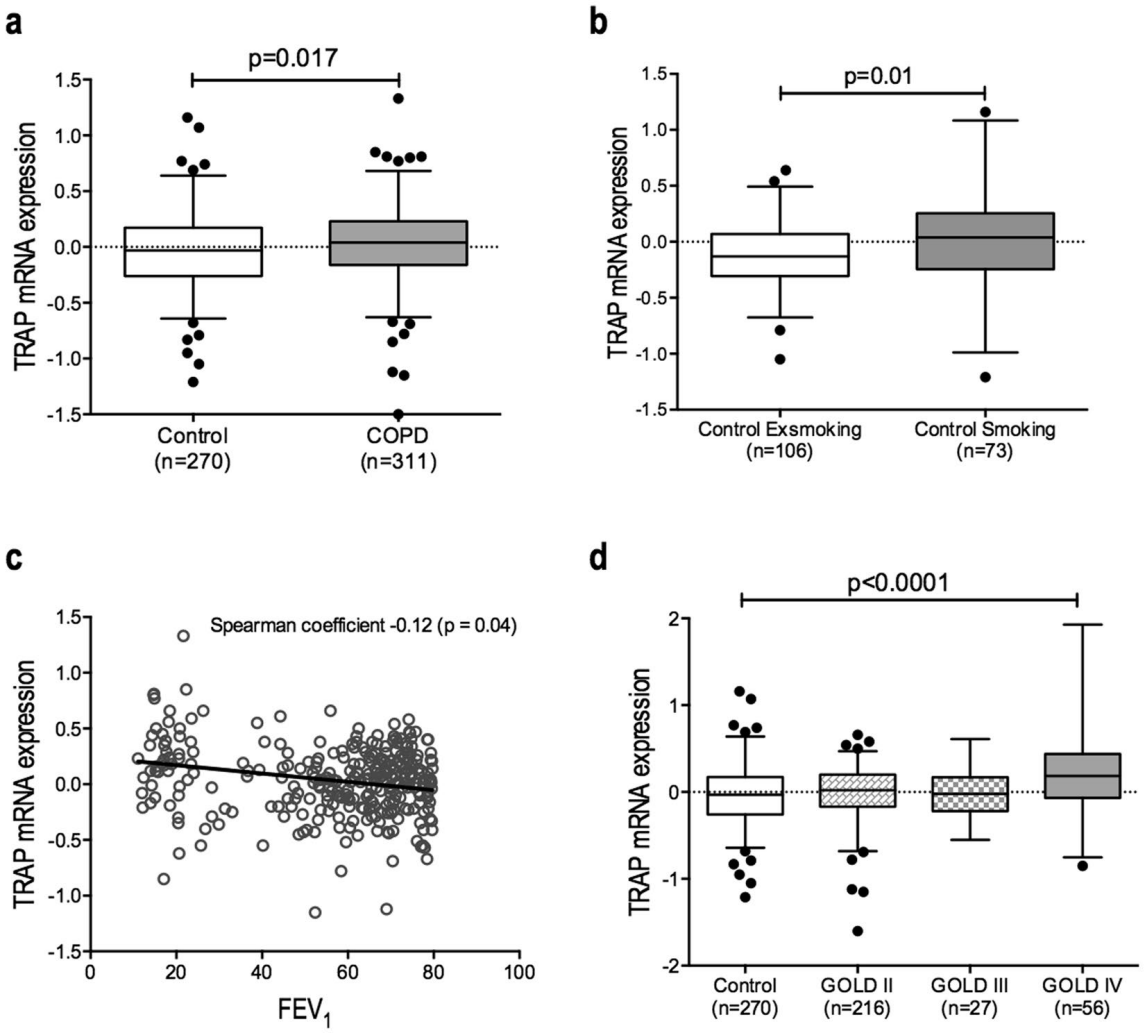
High TRAP expression is associated with COPD. (**a**) TRAP mRNA expression was significantly higher in lung tissue of patients with COPD (n = 311) than in their respective controls (n = 270). (**b**) TRAP mRNA expression was significantly higher in lung tissue of current smoking control individuals (n = 73) than in control exsmokers after at least 5 years of smoking cessation (n = 106). (**c**) TRAP mRNA expression correlated negatively with lung function (FEV_1_). (**d**) TRAP mRNA expression was only significantly higher in lung tissue of patients with severe COPD (GOLD stage IV) compared to control individuals, and not in patients with less severe COPD compared to the controls. Differences between 2 groups were tested with a Mann-Whitney U test, between multiple groups with a Kruskal-Wallis test with a Dunn’s correction for multiple testing. The correlation between TRAP mRNA expression and FEV_1_ was calculated using a Spearman correlation test. A p value smaller than 0.05 was considered significant.

To assess whether TRAP mRNA expression is changed in COPD versus control lung tissue, we did a single gene look-up for TRAP in a genome wide gene expression dataset comparing 311 COPD patients and 270 non-COPD controls^31^. Among the upregulated genes, TRAP was identified as significantly higher in COPD patients compared to control patients (Fig. 2a). To investigate the effect of current smoking on TRAP expression, we additionally compared control individuals currently smoking with individuals that had stopped smoking for at least 5 years in the same dataset. This comparison showed significantly higher expression of TRAP in the individuals that are currently smoking versus ex-smokers (Fig. 2b). A similar analysis among the COPD patients showed no differences between current and ex-smokers (data not shown).

In addition, we examined whether TRAP mRNA expression correlated with lung function in COPD patients (as defined by FEV_1_) and found a significant but weak negative correlation, meaning higher TRAP expression was linked with lower FEV1 values (Fig. 2c). This correlation is mainly caused by the high expression of TRAP in lung tissue of patients with severe COPD: patients with the most severe disease, i.e. highest GOLD stage and therefore lowest FEV1 value, had significantly higher expression of TRAP in lung tissue as compared to nonCOPD controls, while the patients with less severe COPD had similar TRAP expression as compared to controls (Fig. 2d).

### Patients dying of asthma have more TRAP-active macrophages in lung tissue

**Figure 3:**
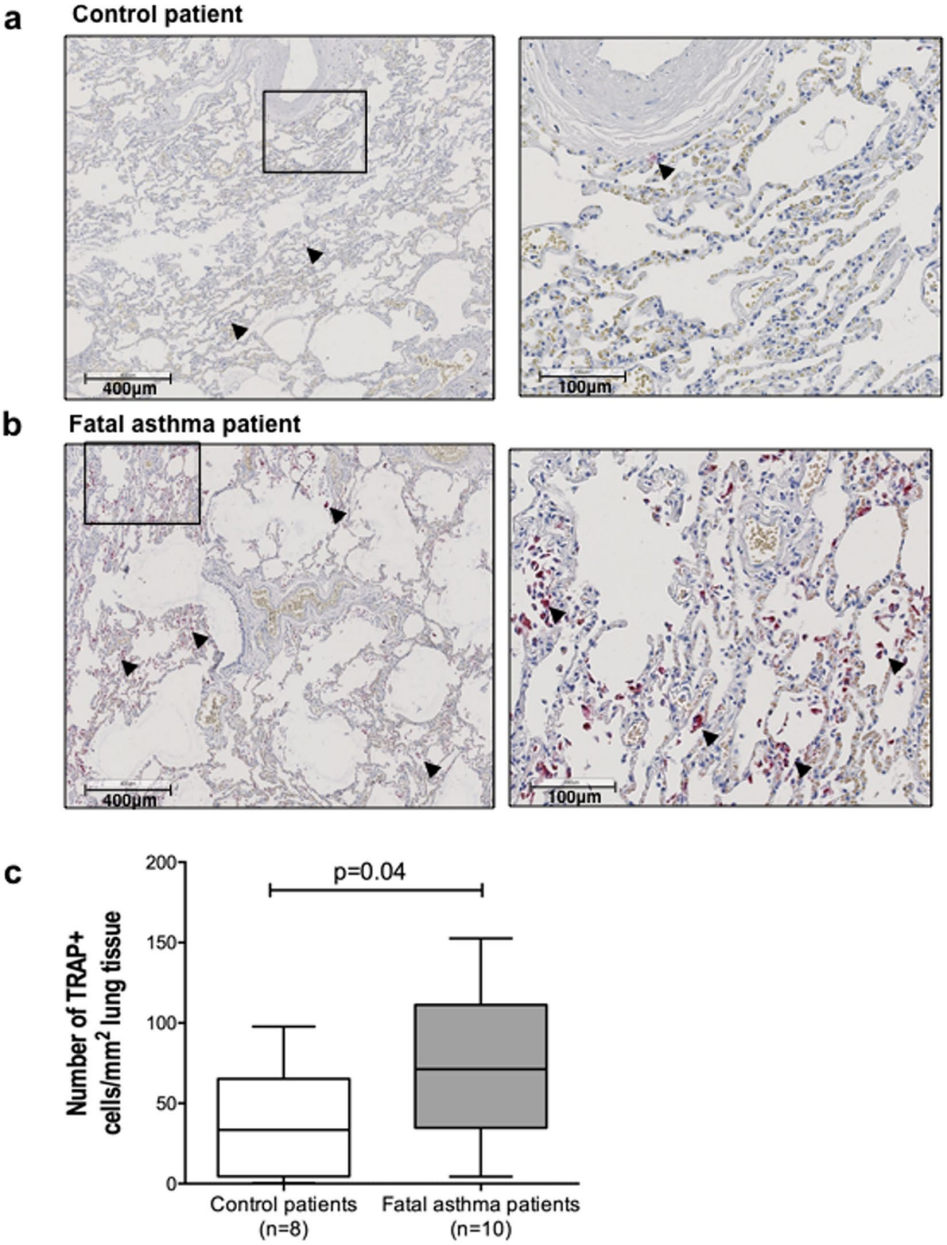
More TRAP-active macrophages are associated with fatal asthma. (**a**) Representative pictures of lung tissue sections of a control individual stained for TRAP activity. Cells positive for TRAP activity (purple) are alveolar macrophages as judged by morphology and tissue location (some indicated by arrows). (**b**) Representative pictures of lung tissue sections of a fatal asthma patient stained for TRAP activity. (**c**) Quantification of the stainings showed that parenchymal lung tissue of patients with fatal asthma (n = 10) contained more TRAP-active macrophages as compared to controls dying of nonpulmonary causes (n = 8). Differences were tested using a Mann-Whitney *U* test. A p value smaller than 0.05 was considered significant.

To assess whether asthma is also characterized by changes in TRAP, we investigated the number of cells staining positive for TRAP activity in lung sections of patients who had died from an asthma attack or had died of non-pulmonary causes. The sections showed that only alveolar macrophages stained positive for active TRAP enzyme, as judged by their morphology and location in the tissue, though not all alveolar macrophages were positive for TRAP activity (Fig. 3a and b, some are indicated by arrows). In addition, the number of macrophages positive for TRAP activity was higher in lung tissue from patients with fatal asthma as compared to control subjects (Fig. 3c).

### The number of TRAP-active cells is higher in mouse models for COPD and asthma

**Figure 4:**
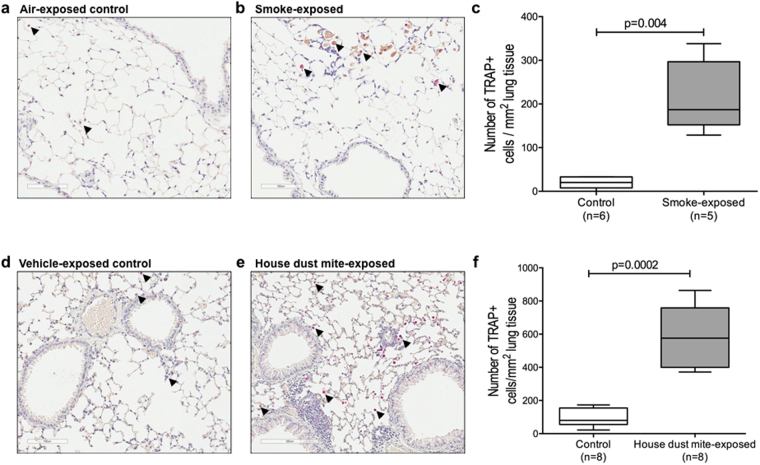
High TRAP activity is associated with exposure to smoke and house dust mite. (**a,b**) Representative pictures of lung tissue sections of an air-exposed control mouse and a smoke-exposed mouse stained for TRAP activity. Alveolar macrophages stained strongly positive for TRAP (purple) as indicated by the arrows. (**c**) Quantification of the stainings showed that parenchymal lung tissue of mice exposed to cigarette smoke for 9 months (n = 5) contained more TRAP-active alveolar macrophages than mice exposed to room air (n = 6). (**d**,**e**) Representative pictures of lung tissue sections of a control mouse and a house dust mite-exposed mouse stained for TRAP activity. Alveolar macrophages stained strongly positive for TRAP (purple) as indicated by the arrows. In lung tissue of HDM-exposed mice faint staining for active TRAP enzyme could also be noticed in inflammatory infiltrates and in epithelial cells of the large airways (**f**) Quantification of the stainings showed that mice exposed to HDM (n = 8) had more TRAP+ alveolar macrophages in parenchymal lung tissue than control mice (n = 8). Differences were tested using a Mann-Whitney *U* test. A p value smaller than 0.05 was considered significant.

To check if higher expression/activity of TRAP in humans with pulmonary disease was a general phenomenon that could be extrapolated to mouse models, we examined TRAP activity in lungs of mice exposed to either cigarette smoke for 9 months (COPD model) or house dust mite (HDM) for 2 weeks (asthma model). Again, we stained for active TRAP enzyme and found that alveolar macrophages, as judged by their morphology and location in the tissue, stained strongly positive for active TRAP enzyme, though not all of them were positive for TRAP activity (Fig. 4a,b,d,e, some are indicated by arrows). In lung tissue of mice that were exposed to cigarette smoke (Fig. 4c or HDM (Fig. 4f) we found significantly more TRAP-positive macrophages than in lung tissue of the relevant control mice. In lung tissue of HDM-exposed mice faint staining for active TRAP enzyme could also be noticed in inflammatory infiltrates and in epithelial cells of the large airways (Fig. 4e).

### TRAP expression is upregulated by RANKL and oxidative stress

**Figure 5:**
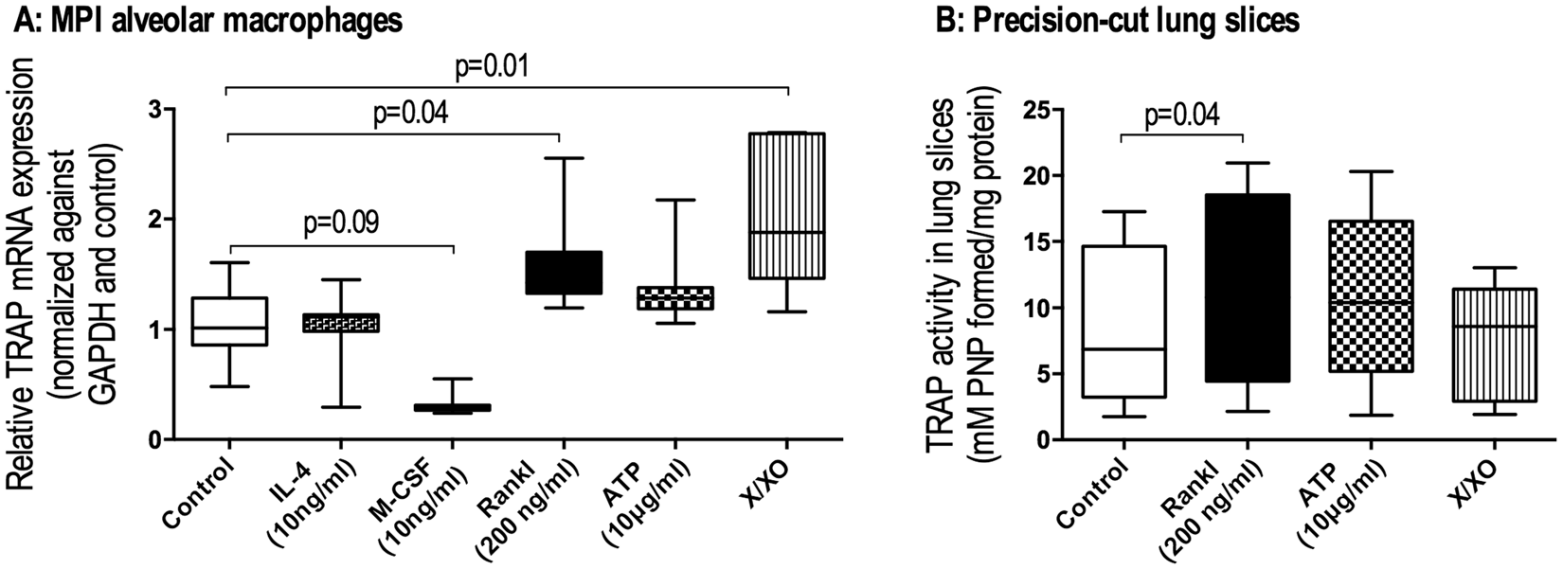
RANKL and oxidative stress are inducers of TRAP. (**A**) TRAP mRNA expression in murine MPI alveolar macrophages was significantly higher after stimulation with 200 ng/ml RANKL (n = 10) for 24 h or 16 h of exposure to xanthine/xanthine oxidase (n = 7) to mimic oxidative stress as compared to control conditions (n = 10). Exposure to 10 ng/ml M-CSF (n = 10) for 24 hrs resulted in a trend towards downregulation of TRAP mRNA expression. 10μg/ml ATP (n = 10) and 10 ng/ml IL-4 (n = 9) had no effect on TRAP mRNA expression. (**B**) TRAP activity was higher in precision-cut murine lung slices incubated with 200 ng/ml RANKL (n = 6) for 24 hrs as compared to slices incubated in control conditions (n = 6). No effect on TRAP activity was seen following stimulation with 10μg/ml ATP (n = 6) or xanthine/xanthine oxidase (n = 6) for 24 hrs. Differences between the multiple groups were tested with a Kruskal-Wallis test with a Dunn’s correction for multiple testing. A p value smaller than 0.05 was considered significant.

In order to study what causes the higher activity and/or expression of TRAP in alveolar macrophages, we exposed murine MPI alveolar-like macrophages (Max Planck Institute, a kind gift from Dr. Gyorgy Fejer^32^) and murine precision-cut lung slices to various stimuli related to COPD and asthma, namely IL-4, M-CSF and RANKL, the damage-associated molecular pattern ATP, and oxidative stress mimicked by the xanthine/xanthine oxidase (X/XO) system. Notably, TRAP mRNA expression in MPI alveolar-like macrophages was significantly higher after stimulation with RANKL and the X/XO system (Fig. 5A). M-CSF stimulation resulted in a trend towards lower TRAP mRNA expression. No significant effects were observed after stimulation with ATP or IL-4.

To study whether changes in mRNA expression would also lead to changes in active enzyme, we used precision-cut lung slices to study the effects of RANKL, ATP and oxidative stress on TRAP activity (Fig. 5B). Only RANKL treatment resulted in significantly higher TRAP activity in lung slices as compared to control conditions. Conversely, ATP treatment and induction of oxidative stress with X/XO treatment did not lead to significant changes in TRAP activity.

### The Au(III) compound AubipyOMe inhibits TRAP activity

Having a potent and specific inhibitor of TRAP can greatly benefit studies into its function, and we therefore investigated whether we could improve on the currently known inhibitors of TRAP^15,20–24^. The most potent inhibitor previously reported is the inorganic complex NaAuCl_4_^20^, but this Au(III) reactive compound is prone to reduction in biological environments and features unspecific protein binding and oxidative damage, which may interfere with many different cellular pathways^33^. Therefore, a series of gold coordination compounds, more stable in biological environments compared to NaAuCl_4_, were evaluated as possible TRAP activity inhibitors: these included mono- and di-nuclear Au(III) compounds with N-donor ligands and the previously tested anti-rheumatic agent sodium aurothiomalate (Myochrysine®, see Supplementary Fig. S1 for the structures of the compounds tested)^20^. The initial screening using commercially bought recombinant TRAP revealed that the compound AubipyOMe possessed the best TRAP inhibition activity described to date, similar to NaAuCl_4_, being able to inhibit the protein activity with IC50 in the nanomolar range (see Supplementary Fig. S2 for inhibition curves of all compounds tested).

**Figure 6:**
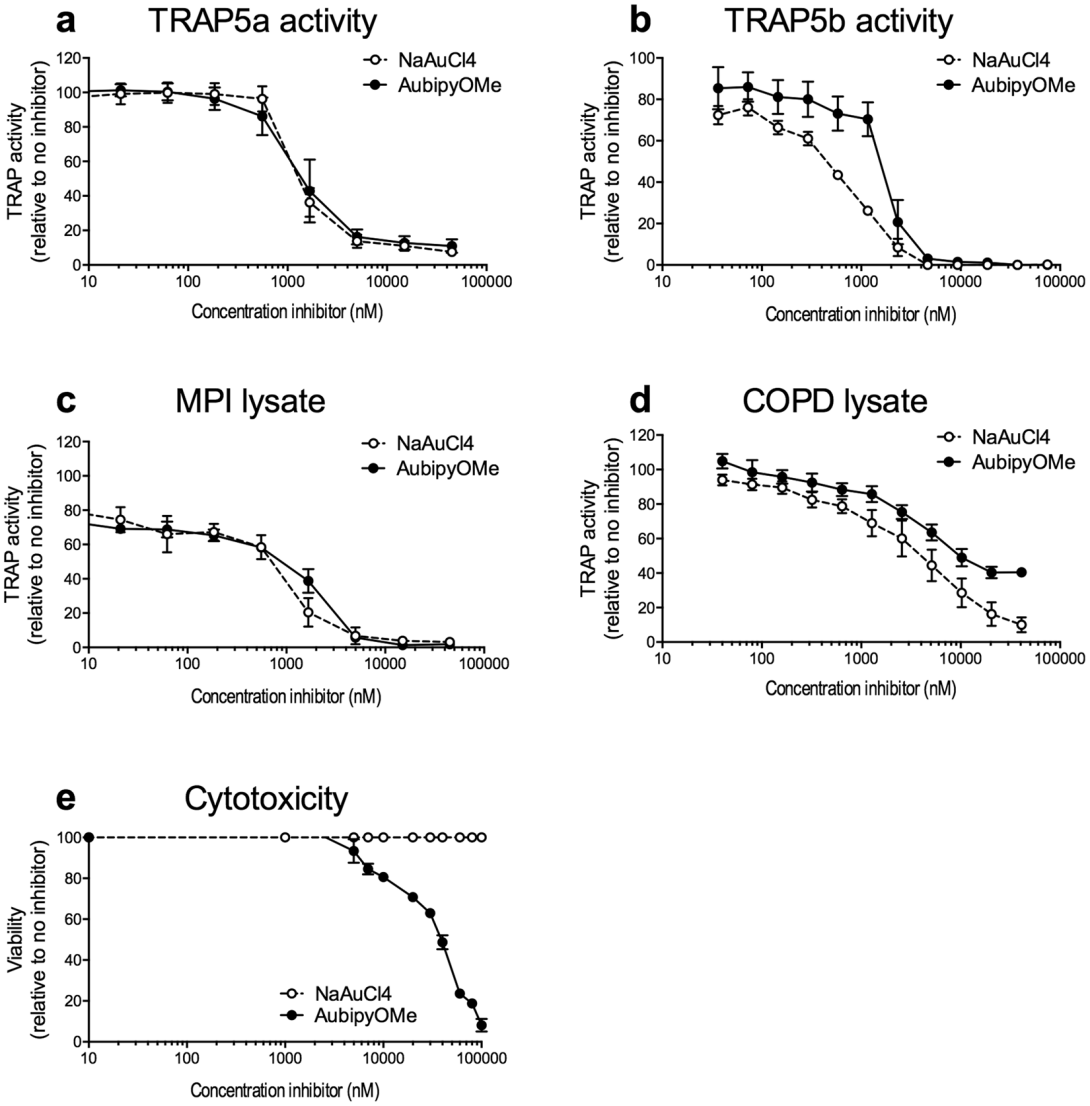
AubipyOMe is a potent TRAP inhibitor. (**a**) Activity of recombinant TRAP5a was inhibited by NaAuCl_4_ (IC50: 1.4 ± 0.2 μM) and AubipyOMe (IC50: 1.3 ± 0.5 μM) (n = 3). (**b**) Activity of recombinant TRAP5b was inhibited by NaAuCl_4_ (IC50: 1.0 ± 0.2 μM) and AubipyOMe (IC50: 1.8 ± 0.3 μM) (n = 3). (**c**) TRAP activity in lysates of murine MPI alveolar macrophages was inhibited by NaAuCl_4_ (IC50: 0.7 ± 0.0 μM) and AubipyOMe (IC50: 1.7 ± 0.4 μM) (n = 3). (**d**) TRAP activity in lysates from lung tissue of COPD patients was inhibited by NaAuCl_4_ (IC50: 4.8 ± 1.3 μM) and AubipyOMe (IC50: 3.6 ± 0.0 μM) (n = 3). (**e**) Incubation of RAW264.7 macrophages with AubipyOMe only inhibits cell viability at concentrations far exceeding the IC50 value, NaAuCl_4_ did not have any toxicity (n = 4).

Thus, we continued our investigations with AubipyOMe, and NaAuCl_4_ as reference compound, to further assess its selectivity for the TRAP isoforms 5a and 5b. AubipyOMe inhibited TRAP5a activity with an IC50 value of 1.3 ± 0.5 μM and TRAP5b with an IC50 value of 1.8 ± 0.3 μM (Fig. 6a and b). These IC50 values were comparable to the values found for NaAuCl_4_ (see Table 1 and Supplementary Fig. S3 for the individually fitted curves used to calculate IC50 values). To assess the inhibition potencies in more relevant biological settings, we continued testing AubipyOMe and NaAuCl_4_, in cell and tissue lysates. TRAP activity in cell lysates of MPI alveolar macrophages was significantly inhibited in the presence of AubipyOMe and NaAuCl_4_ with IC50 values of 1.7 ± 0.4 μM and 0.7±0.0 μM, respectively (Fig. 6c, Table 1, and Supplementary Fig. S3 for the individually fitted curves used to calculate IC50 values). Importantly, the inhibitory effects of AubipyOMe and NaAuCl_4_ were also tested on TRAP activity in pooled lung tissue lysates from COPD patients. AubipyOMe significantly inhibited TRAP activity in these lysates with an IC50 value of 4.8 ± 1.3 μM, while NaAuCl_4_ inhibited the activity with an IC50 value of 3.6 ± 0.0 μM (Fig. 6d, table, and Supplementary Fig. S3 for the individually fitted curves used to calculate IC50 values). At concentrations around these IC50 values, both AubipyOMe and NaAuCl_4_ had no cytotoxic effects on RAW264.7 macrophages (Fig. 6e). Only in very high concentrations AubipyOMe showed some cytotoxicity (IC50 around 35 μM) and NaAuCl_4_ did not display significant toxicity (IC50 > 200 μM).

**Table 1:** Effect of AubipyOMe and NaAuCl_4_ on TRAP activity (data are represented as mean ± standard error).

Compounds	IC50 (µM)			
	TRAP5a	TRAP5b	MPI	COPD
NaAuCl_4_	1.4 ± 0.2	1.0 ± 0.2	0.7 ± 0.0	4.8 ± 1.3
AubipyOMe	1.3 ± 0.5	1.8 ± 0.3	1.7 ± 0.4	3.6 ± 0.0

### Macrophage migration depends on TRAP activity and is inhibited by AubipyOMe

**Figure 7:**
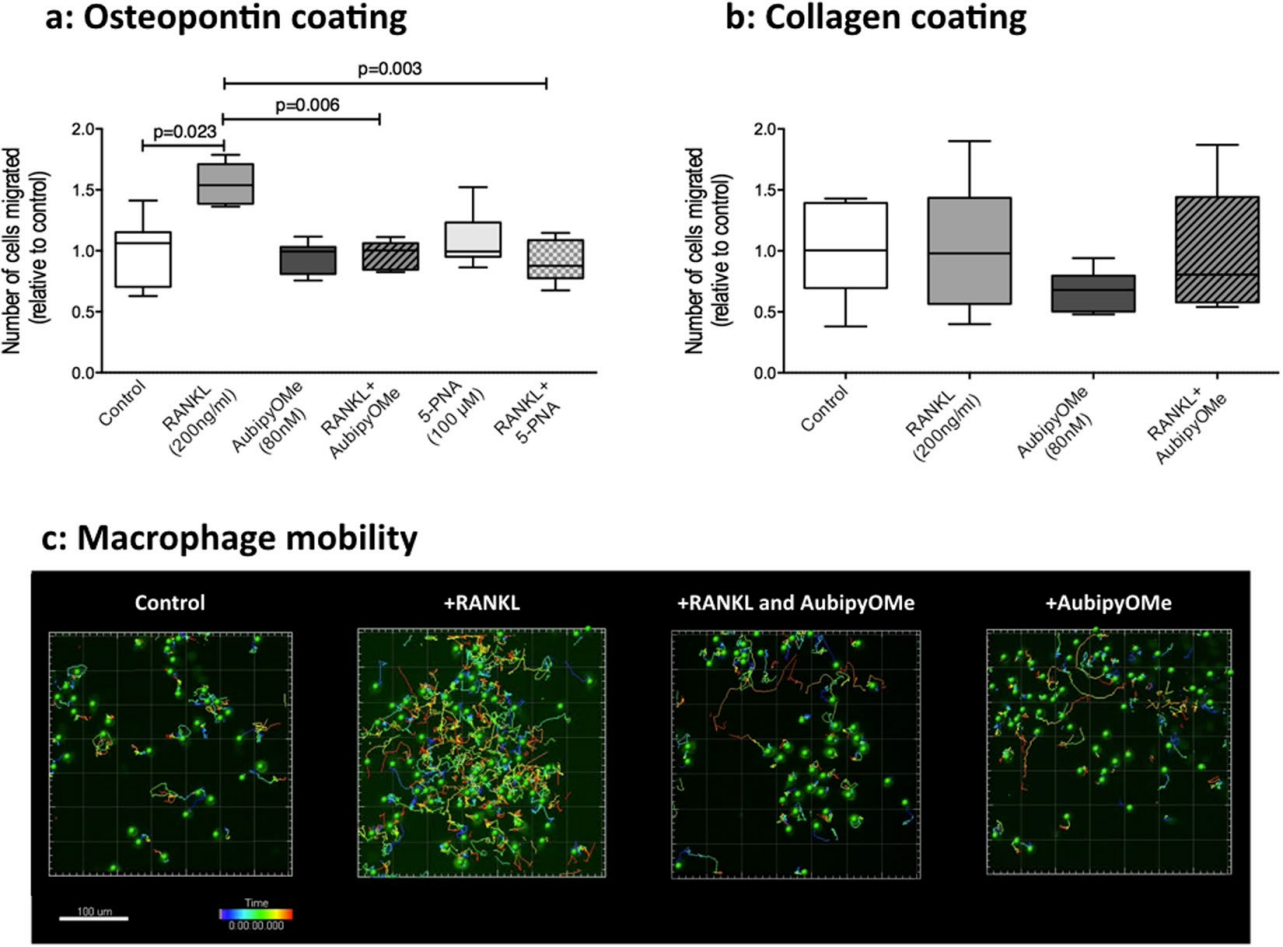
TRAP is involved in macrophage migration. (**a**) In a transwell set-up, RANKL-stimulated RAW264.7 macrophages (200 ng/ml) for 72 h migrated significantly more through an osteopontin-coated membrane as compared to control macrophages. This RANKL-induced migration was not seen in the presence of our newly proposed TRAP inhibitor AubipyOMe (80 nM) or in the presence of previously published TRAP inhibitor 5-PNA (100 μM). Both inhibitors did not affect migration on their own. Data represent seven independent experiments. Differences between the multiple groups were tested with a Kruskal-Wallis test with a Dunn’s correction for multiple testing. A p value smaller than 0.05 was considered significant (**b**) Using collagen-coated membranes, no differences were found in transwell migration when RAW264.7 macrophages were stimulated with RANKL (200 ng/ml) or not and no effect of TRAP inhibitor AubipyOMe (80 nM) on migration was found. Data represent six independent experiments. Differences between the multiple groups were tested with a Kruskal-Wallis test with a Dunn’s correction for multiple testing. A p value smaller than 0.05 was considered significant (**c**) Live cell tracking of macrophages in osteopontin-coated wells revealed that macrophage migratory behavior was higher in the presence of RANKL (200 ng/ml) as compared to control and AubipyOMe (80 nM) inhibited this migratory behavior (movies can be found in the online supplementary information, Movies 1–4).

Osteoclast migration was previously shown to be TRAP-dependent through the ability of TRAP to dephosphorylate osteopontin^2,14^. To investigate if this is also the case for macrophages we used RAW264.7 macrophages because we could modulate TRAP expression and activity from low to high by pretreatment with RANKL in these cells^34^. We subsequently investigated the effects of having TRAP activity and inhibition of this TRAP activity on macrophage migration in a transwell and live cell-imaging setup (Fig. 7).

RAW264.7 macrophages pretreated with RANKL for 72 hours migrated significantly more through an osteopontin-coated membrane as compared to unstimulated cells (Fig. 7a). This effect was osteopontin-specific, as migration over a membrane coated with collagen was not affected by RANKL pretreatment (Fig. 7b). RANKL pretreatment and the presence of our proposed TRAP inhibitor AubipyOMe led to significantly less migration of RAW264.7 macrophages as compared to RANKL pretreatment alone. Accordingly, the presence of the previously published TRAP-inhibitor 5-PNA, that inhibits TRAP-dependent migration of TRAP-overexpressing cancer cells also inhibited macrophage migration induced by RANKL pretreatment^15^. Treatment of the cells with either inhibitors alone did not affect macrophage migration and neither did AubipyOMe affect migration when cells were grown on collagen-coated membranes, indicating that the inhibition was not unspecific (Fig. 7).

Furthermore, we investigated the involvement of TRAP in macrophage migration using live cell imaging. RAW264.7 macrophages plated on wells coated with osteopontin showed more migratory behavior after RANKL pretreatment than control cells not pretreated with RANKL (Fig. 7c, movie 1 versus 2 in supplementary information). The observed increased migratory behavior was not recorded when RANKL-pretreated cells were in the presence of AubipyOMe (Fig. 7c, movie 3, supplementary information). The presence of AubipyOMe did not affect the migratory behavior of control cells (Fig. 7c, movie 4, supplementary information).

For both the transwell migration experiments as well as the live cell imaging, pretreatment with RANKL for 48–72 hours did not lead to the development of multinucleated osteoclast-like cells. This can also be appreciated from the images in Fig. 7c and the movies in the supplementary information available.

## Discussion

A distinct characteristic of alveolar macrophages is the high expression of TRAP, yet the function of this enzyme in the lung is unclear. We first set out to investigate whether the presence of TRAP is changed in obstructive pulmonary diseases to see if it has any relevance to these disease processes. We observed higher TRAP expression in COPD and asthma as compared to control and we found that this increase may be triggered by RANKL and/or oxidative stress, though the functional consequences of this higher expression and activity remain to be determined. To aid the elucidation of the role of TRAP in the lung, we developed a new type of TRAP-inhibitor: AubipyOMe. This Au(III)-based compound is the most potent inhibitor of the phosphatase function of TRAP described to date and was used here to show that TRAP appears to be involved in macrophage migration.

To the best of our knowledge, TRAP expression levels have never been studied before in relation to COPD. Only three studies have looked at the effect of smoking, the most important risk factor of COPD, and found elevated TRAP mRNA and/or protein expression in smokers^17–19^. Our results confirm these previous findings for current smoking and we now also show that, TRAP mRNA expression is higher in lung tissue of COPD patients compared with controls, independent of smoking status. Since TRAP mRNA expression is higher with the highest disease severity, a disease-specific factor may be causing the increase in TRAP expression on top of smoking-related induction of TRAP.

TRAP has not been studied in the context of asthma. This chronic lung disease has a different pathogenesis from COPD but we now show that it is also characterized by higher numbers of TRAP-active macrophages in lung tissue as compared to controls. Hence, we investigated some overlapping cytokines and conditions of both obstructive lung diseases to identify the cause of the high TRAP expression/activity in these lung diseases. *In vitro* experiments showed that the most likely candidate to induce TRAP expression/activity is RANKL. RANKL is a well-known inducer of TRAP expression in osteoclasts (multinuclear bone macrophages) and was included as a positive control. However, higher levels of RANKL have also shown to be present in patients with COPD, especially those suffering from osteoporosis, a well-known comorbidity of emphysematous COPD^35,36^. Therefore, the high levels of circulating RANKL may explain the high TRAP expression and activity in lung tissue of COPD patients. Our data showing that especially GOLD stage IV patients, transplanted for severe emphysema, have the highest mRNA expression of TRAP are in line with this observation. To the best of our knowledge, no reports have been published about the levels of RANKL in (fatal) asthma patients and it therefore remains unclear whether and how RANKL could play a role in (fatal) asthma.

Our results also showed that TRAP mRNA expression is higher in macrophages cultured under high-oxidative-stress conditions. Indeed, oxidative stress is a known inducer of osteoclast formation accompanied by increased TRAP5b expression in these cells^37,38^. Patients with COPD have been shown to have high levels of oxidative and nitrosative stress in their lungs and in asthma basal oxidative stress levels are elevated as a result of chronic inflammation^39–42^. This oxidative stress may therefore be responsible for elevated macrophage TRAP mRNA expression. Interestingly, the higher TRAP *expression* following oxidative stress in macrophages *in vitro* did not result in higher TRAP *activity* in lung slices that were cultured under similar conditions. The reason for this conflicting result is unclear but may relate to the activity assay not being sensitive enough to pick up differences in lung slices or to the slicing and subsequent incubation of lung tissue, leading to abnormal consumption of reducing agents like glutathione in the tissue that are necessary for optimal TRAP activity^43,44^.

TRAP itself may also contribute to oxidative stress levels in the lung through its oxygen radical producing potential^44^. In fact, alveolar macrophages are an important first-line defense against pathogens and their TRAP expression was shown to contribute to ROS formation and bacterial killing^3,16,45,46^. The monomeric, intracellular TRAP5a isoform can generate cellular oxidative stress through oxidation of one of the iron atoms in its active site^3^. High TRAP expression/activity in COPD and asthma may therefore contribute to the increased levels of oxidative stress found in these diseases^39–42^.

To further aid investigations into the function of (elevated) TRAP activity in alveolar macrophages, we identified Au(III)-containing compounds as potent inhibitors of TRAP, with AubipyOMe as the most active one described to date^15,20–24^. It should be noted that this compound (and analogues) has previously been tested for its reactivity with other proteins, but the inhibitory effects were extremely moderate compared to those shown here for the TRAP isoforms^47,48^. Interestingly, in contrast to the other previously characterized inhibitor 5-PNA that only inhibits TRAP5b, AubipyOMe could inhibit both isoforms to a similar extent^15^. Docking studies combined to molecular modeling will have to elucidate which parts of the molecules are responsible for the differential effects. These results are an excellent basis for further rational design of compounds with high affinity and high inhibition potential of TRAP and its specific isoforms.

To test our inhibitor in a physiological setting with a physiological substrate we investigated whether TRAP could be involved in migration of macrophages by dephosphorylation of osteopontin as has been reported before for osteoclasts and cancer cells^2,14,15,49,50^. Extracellular TRAP5b activity was shown to contribute to osteoclast migration by dephosphorylation of osteopontin thereby reducing (α_v_β_3_-integrin-mediated) cell adhesion of osteoclasts. We now show that TRAP activity in macrophages has a similar function, because upregulation of TRAP by RANKL stimulation resulted in more macrophage migration over an osteopontin-coated membrane. In addition, subsequent inhibition of TRAP activity by AubipyOMe, as well as the previously reported TRAP-inhibitor 5-PNA, resulted in less macrophage migration, implying a similar mode of action of these two inhibitory compounds. As 5-PNA only inhibits TRAP5b, the observed migratory behavior is likely to be dependent on this isoform.

Functional consequences of enhanced macrophage migration in lung tissue in diseases like COPD and asthma remain to be investigated. Work of the group of Väänänen *et al*. showed that TRAP is involved in matrix degrading processes by assisting in trafficking of collagens in vesicles through the cell and that TRAP colocalized with phagocytosed material within alveolar macrophages^16,51^. Therefore, alveolar macrophage TRAP may participate in the tissue remodeling processes that play a role in both asthma and COPD.

Another possible role for TRAP in COPD and asthma could be the regulation of interferon alpha (IFNα) production. Both COPD and asthma are characterized by exacerbations, often induced by viral infections^52^. IFNα is important in defenses against viruses and Briggs *et al*. recently showed that TRAP inhibits IFNα production by regulating intracellular levels of phosphorylated osteopontin in dendritic cells^53^. Expression of nonfunctional TRAP led to higher levels of IFNα and therefore high expression of TRAP (like in COPD and asthma) may result in a lower IFNα production. Indeed, lower levels of IFNα have been detected in patients with asthma and COPD^54,55^. Based on these considerations, the gold-based TRAP inhibitor AubipyOMe could be used to investigate the role of TRAP in IFNα production by alveolar macrophages.

An unexpected outcome was the clear downregulation of TRAP expression in alveolar-like macrophages after M-CSF stimulation. In the bone field, previous studies have shown that M-CSF is necessary for osteoclast formation and TRAP expression^56^, but exposure to high levels of M-CSF early during differentiation may actually blunt differentiation into osteoclasts and therefore TRAP expression^57,58^. A similar mechanism may be in play here. In addition, this discrepancy in M-CSF responsiveness may be caused by the fact that we used alveolar-like macrophages, which are derived from fetal monocytes/yolk sac macrophages and self-maintain in lung tissue during life, while osteoclasts are derived from hematopoietic stem cells and are replenished from bone marrow^59–63^. Since alveolar macrophages are particularly dependent on GM-CSF for their development, this could explain the discrepancy in the response to M-CSF^61^.

In conclusion, TRAP expression and activity are high in COPD and fatal asthma and in relevant mouse models. One of the roles of TRAP may be to facilitate macrophage migration, but the consequences of this for the pathogenesis of COPD and asthma are still unclear. The development of our potent gold-based TRAP inhibitor now allows more detailed studies into the function of TRAP in the lung and diseases of the lung characterized by higher TRAP activity such as asthma and COPD.

## Materials and Methods

All methods were carried out in accordance with relevant national and local guidelines and regulations regarding the use of experimental animals, tissues of human subjects and proper research conduct. More detailed information for each part is available in the online supplementary information “Material and Methods” section.

### Human tissue

#### COPD

Gene expression data of TRAP was obtained from a large gene expression study comparing lung tissue from 311 patients with COPD and 270 non-COPD controls that were part of the Lung eQTL consortium. Details of this population can be found in Supplementary Table S1. All lung tissue samples were obtained in accordance with Institutional Review Board guidelines at the three sites: Laval University (Quebec, Canada), University of British-Columbia (Vancouver, Canada) and Groningen University (Groningen, The Netherlands). All patients provided written informed consent and the study was approved by the ethics committees of the Institut universitaire de cardiologie et de pneumologie de Québec and the UBC-Providence Health Care Research Institute Ethics Board for Laval and UBC, respectively. The study protocol was consistent with the Research Code of the University Medical Center Groningen and Dutch national ethical and professional guidelines (“Code of conduct; Dutch federation of biomedical scientific societies”; http://www.federa.org). A detailed description of the whole genome mRNA profiling has been previously published by Brandsma *et al*. and Hao *et al*.^31,64^.

#### Asthma

Post mortem lung tissues from subjects with fatal asthma or subjects who died from nonpulmonary causes (controls) were retrieved from the Department of Pathology of São Paulo University (São Paulo, Brazil). Patient characteristics can be found in Supplementary Table S2. A detailed clinical and demographic description of this population has been previously published by Mauad *et al*.^65^. Diagnosis was confirmed by macro- and microscopic examination at autopsy and by an interview with the next of kin. Written informed consent was obtained with the next of kin. All experimental protocols within this study were approved by the institutional ethics committee Comissão de Ética para Análise de Projetos de Pesquisa - CAPPesq do Hospital das Clínicas, São Paulo University Medical School and were carried out in accordance with their guidelines. For this study we investigated the presence of TRAP activity in paraffin-embedded peripheral lung tissue samples of 10 asthma patients and 8 controls as described below.

### Animal experiments

During the experiments, all animals were held under specific pathogen-free conditions in groups of 4–6 mice per cage in a temperature-controlled room with a 12h dark/light cycle and permanent access to food and water. The Groningen University Institutional Animal Care and Use Committee approved these experiments according to strict governmental and international guidelines on animal experimentation (DEC2857, DEC5318, and DEC6416AA-001).

#### Smoke-induced lung inflammation

To model COPD, we exposed five male A/JOlaHsd mice (Harlan, Horst The Netherlands, 8–10 weeks old) nose-only to mainstream cigarette smoke for 9 months in an experimental set-up as described before by us^66^. Mice were sacrificed after 9 months and lungs were collected, formalin-fixed and embedded in paraffin for histological analysis of TRAP activity.

#### Allergic lung inflammation

To model asthma, we exposed male and female BALB/c mice (Harlan, Horst The Netherlands, 8–10 weeks old) intranasally to whole body house dust mite (HDM) extract (*Dermatophagoides pteronyssinus*, Greer laboratories, Lenoir, USA) in 40 μl phosphate-buffered saline (PBS) according to a protocol we have described before^67^. Mice were sacrificed on day 24 and lungs were collected for histological analyses and TRAP activity analyses. Other parameters of allergic lung inflammation of these animals are described in detail in our previous publication^67^.

### Enzyme histochemistry for TRAP activity

Presence of active TRAP was assessed using a histochemical method relying on conversion of chromogen Fast Red by active TRAP (see online supplement). The number of positive alveolar macrophages (based on morphology and tissue location) was counted manually with the aid of ImageScope software (Leica Biosystems, Son, The Netherlands) in human and murine lung tissue sections (on average a surface of 9 mm^2^ was measured of each section) and corrected for the surface area of the corresponding lung tissue.

### Cell culture of macrophages

Self-propagating murine alveolar-like macrophages (MPI macrophages, a kind gift from dr. G. Fejer, Plymouth University, Devon, UK) were cultured in RPMI 1640 medium (Gibco, Bleiswijk, The Netherlands) as described by Fejer *et al*.^32^. Cells were stimulated with a superoxide-generating system (0.2 mM Xanthine + 10 mU/ml Xanthine oxidase (Sigma-Aldrich, Zwijndrecht, The Netherlands)) for 16 hrs to mimic oxidative stress, or for 24 hrs with the damage-associated molecular pattern ATP (1, 10 or 100 μg/ml, Sigma-Aldrich), RANKL (200 ng/ml, produced and provided by dr. R.H. Cool, University of Groningen, The Netherlands^68^), IL-4 (10 ng/ml, Peprotech, Rocky Hill, USA), or M-CSF (10 ng/ml, Peprotech). Cells were harvested for mRNA isolation purposes.

RAW264.7 macrophages (American Type Culture Collection) were cultured in Dulbecco’s modified Eagle’s medium (Invitrogen, The Netherlands). RAW264.7 macrophages were used in transwell and cell-tracking experiments, as further explained in the section “Inhibition of macrophage migration by AubipyOMe”.

### Quantitative Real-Time PCR

The following primers were used to determine TRAP mRNA expression, Primers used for RT-PCR were obtained from Sigma-Aldrich: TRAP forward: 5′-GCTGTCCTGGCTCAAAAAGC-3′; TRAP reverse: 5′-CACACCGTTCTCGTCCTGAA-3′; GAPDH forward: 5′-ACAGTCCATGCCATCACTGC-3′; GAPDH reverse: 5′-GATCCACGACGGACACATTG-3′. For each sample, the threshold cycles (Ct values) were calculated with the SDS 2.3 software program (Applied Biosystems) and mRNA expression was normalized against GAPDH. Experiments were repeated at least four times.

### Precision-cut lung slices

Lungs of male C57BL/6 mice (20–30 gr) of in total six mice were used to make precision-cut lung slices. Lung slices, diameter 5-mm and weight ± 5 mg, were prepared with a Krumdieck tissue slicer (Alabama Research and Development, Munford, USA) as described by us before for liver slices^69^. After slicing, murine lung slices were transferred to 12-well plates with pre-warmed DMEM + Glutamax medium (1.3 ml + supplements) and incubated in triplicate with the following stimulants: vehicle, RANKL (200 ng/ml), ATP (10 μg/ml), or Xanthine (0.2 mM) + Xanthine oxidase (10 mU/ml). Three slices of each condition were pooled and used to measure TRAP activity.

### TRAP activity assay on lysates of MPI macrophages and precision-cut lung slices

TRAP activity levels were determined in lung slice homogenates or MPI macrophage lysates by incubation at 37 °C for 1 hour with an L-para-Nitrophenylphosphate (PNPP) solution [100 mM PNPP, 200 mM sodium citrate, 200 mM sodium chloride, 80 mM sodium tartrate, pH 4.5] at a 1:1 ratio. Absorption at 410 nm, with 490 nm as a reference value, was measured using a spectrophotometer. Each sample was measured in duplicate and stimulus outcome was calculated relative to the nonstimulated control absorption level.

### Recombinant TRAP preparations and proteolytic digestion of TRAP

Recombinant unspecified human TRAP was purchased from R&D (Minneapolis, USA). Recombinant human TRAP5a and 5b were produced and purified according to a protocol based on several sources^70–72^ using an ÄKTApurifier™ 10 FPLC system (GE Healthcare, Danderyd, Sweden) as previously described^15^.

### Identification of TRAP inhibitors

Initially, a small library of gold compounds was tested for TRAP inhibition using a TRAP activity assay with recombinant unspecified human TRAP (R&D). The Au(III) compounds [Au(terpy)Cl]Cl_2_ (terpy = terpyridine, Auterpy), [Au_2_(*μ*-O)_2_(bipy)_2_](PF_6_)_2_ (bipy = 2,2′-bipyridine, Auoxo) and [Au(bipyOMe)Cl_2_][PF_6_] (bipyOMe = 4,4′-dimethoxy-2,2′-bipyridine, AubipyOMe) were synthesized as previously described and their purity was confirmed by elemental analysis and showed to be >98%^47,73,74^. The anti-rheumatic Au(I) compound sodium aurothiomalate and the reference Au(III) complex NaAuCl_4_ were purchased from Sigma-Aldrich.

Inhibitor dilutions were prepared in acetate buffer from freshly prepared stock solutions (10 mM in DMSO). Recombinant unspecified TRAP (1.25 ng/ml, pH 4.5), TRAP5a (150 ng/mL, pH 5) or TRAP5b (150 ng/mL, pH 5.8) were incubated at 37 °C for 30 minutes with PNPP solution [10 mM PNPP, 200 mM sodium acetaat, 300 mM potassium chloride) at the indicated pH at a 1:1 ratio and increasing concentrations of NaAuCl_4_ (range 0–40 μM) or Au compounds (range 0–5.1 μM). To stop the reaction, 1M NaOH was added and absorption at 410 nm, with 490 nm as a reference value, was measured using a spectrophotometer.

#### Testing of inhibitors on cell and tissue lysates

Mouse alveolar macrophage lysates were obtained by resuspending 500.000 MPI macrophages in 300 μl acetate buffer. Human lung lysates from COPD patients were used to test the inhibitor on human TRAP present in lung tissue. Lysates from in total 18 COPD patients were pooled (see Supplementary Table S3 for patient characteristics).

AubipyOMe was tested in the range 0–40 μM with MPI cell lysates or pooled lung tissue lysates from COPD patients using a TRAP activity assay. Lysates were incubated at 37 °C for 30 min in the presence of PNPP solution (1:1 ratio). 1M NaOH was added to stop the reaction and absorption was measured at 410 nm with 490 nm as a reference value.

### IC50 calculations

The inhibitory effects of gold compounds were calculated as the ratio of absorbance between the treated and untreated wells. The IC50 values were calculated using nonlinear curve fitting with a variable slope in Graphpad Prism 6 (Graphpad Software, la Jolla, USA). An average IC50 of three independent experiments was calculated.

### MTT assay

The effect of AubipyOMe and NaAuCl_4_ on RAW264.7 cell growth was assessed with a classical MTT assay. The IC50 values were calculated using nonlinear curve fitting with a variable slope in Graphpad Prism 6 (Graphpad Software) An average IC50 of four independent experiments was calculated.

#### Transwell experiment to assess macrophage migration

Migration of RAW264.7 macrophages was assessed using a transwell-culturing system with inserts (Sigma-Aldrich) coated with osteopontin (10 μg/ml, R&D). Bovine collagen-coated inserts (10 μg/ml, Advanced Biometrix, Carlsbad, USA) were used as matrix control. Cells were incubated with or without RANKL (200 ng/ml) for 72 hours to induce TRAP activity or not^75^, in the presence or absence of AubipyOMe (80 nM) in quadruplicate. The previously described TRAP inhibitor 5-PNA (100 μM) was used as selective positive control for TRAP inhibition^15,23^. The number of cells migrated to the lower well, including dead cells, was calculated relative to control cells and individual experiments were done at least six times.

#### Confocal imaging of macrophage migration

Briefly, RAW264.7 macrophages were plated on osteopontin-coated (10 μg/ml, R&D) Lab-tek chamber slides (Nunc, Hatfield, USA). Cells were incubated with or without RANKL (200 ng/ml) for 48 hours to induce TRAP activity or not^75^, followed by labeling with carboxyfluorescein diacetate succinimidyl ester (CFSE, Invitrogen, Life Technologies Europe BV, Bleiswijk, The Netherlands) to visualize cells for live cell imaging using a confocal microscope (Solamere Nipkow Confocal Live Cell Imaging system, Solamere Technology Group, Salt lake city, USA). Cell movement was tracked overnight in the presence of 1% zymosan solution (Sigma-Aldrich), to stimulate cell movement, and with or without TRAP inhibitor AubipyOMe (80 nM). Pictures were taken every 10 min and were transformed into movies with Image J and Imaris x64 (Bitplane, Zurich, Switzerland) software^76^.

### Statistical analysis

All data were assumed to have nonnormal distributions. Statistical differences between two groups were calculated using a Mann-Whitney *U* test. When comparing multiple groups, a Kruskal-Wallis with a Dunn’s correction for multiple testing was performed. When studying correlations, a Spearman coefficient was calculated to test for significant relationships. P < 0.05 was considered significant for all data. Statistical tests were done using Graphpad Prism 6 (Graphpad Software). Data are presented as Box and Whiskers plots with the whiskers representing the 2.5–97.5 percentile.

### Data availability statement

The datasets generated during and/or analysed during the current study are available from the corresponding author on reasonable request.

## Electronic supplementary material

Movie 1

Movie 2

Movie 3

Movie 4

Supplementary info file
