# Supplementary material for: A Potent Tartrate Resistant Acid Phosphatase Inhibitor to Study the Function of TRAP in Alveolar Macrophages
Source: Sci Rep. 2017 Oct 3;7:12570. doi: 10.1038/s41598-017-12623-w (PMC5626781; doi:10.1038/s41598-017-12623-w)
Supplement: Supplementary file 5 — Supplementary info file [file 41598_2017_12623_MOESM5_ESM.pdf]

Supplementary information of:

A Potent Tartrate Resistant Acid Phosphatase Inhibitor to Study the Function of TRAP in Alveolar Macrophages

Carian E Boorsma[1,8#], T Anienke van der Veen, [1,8#] Kurnia SS Putri[2], Andreia de Almeida[3], Christina Draijer[1,8], Thais Mauad[4], Gyorgy Fejer[5], Corry-Anke Brandsma[6,8], Maarten van den Berge[7,8], Yohan Bossé[9], Don Sin[10,11], Ke Hao[12,13,14], Anja Reithmeier[15], Göran Andersson[15], Peter Olinga[2], Wim Timens[6,8], Angela Casini[1,3], Barbro N Melgert[1,8*]

Materials and Methods

Detailed information regarding the materials and methods used in the manuscript "A Potent Tartrate Resistant Acid Phosphatase Inhibitor to Study the Function of TRAP in Alveolar Macrophages". All methods were carried out in accordance with relevant national and local guidelines and regulations regarding the use of experimental animals, tissues of human subjects and proper research conduct.

Human tissue

COPD

Gene expression data of TRAP was obtained from a large gene expression study comparing lung tissue from 311 patients with COPD and 270 non-COPD controls that were part of the Lung eQTL consortium. Details of this population can be found in supplemental table S1. Lung tissue samples were derived from patients with or without COPD undergoing lung tumor resection surgery or lung transplantation for severe COPD. In case of tumor resections, macroscopically nontumorogenic lung tissue was taken far distant from the tumor and histology of all samples was checked for abnormalities using standard haematoxylin and eosin staining. All lung tissue samples were obtained in accordance with Institutional Review Board guidelines at the three sites: Laval University (Quebec, Canada), University of British-Columbia (Vancouver, Canada) and Groningen University (Groningen, The Netherlands). All patients provided written informed consent and the study was approved by the ethics committees of the Institut universitaire de cardiologie et de pneumologie de Québec and the UBC-Providence Health Care Research Institute Ethics Board for Laval and UBC, respectively. The study protocol was consistent with the Research Code of the University Medical Center Groningen and Dutch national ethical and professional guidelines ("Code of conduct; Dutch federation of biomedical scientific societies"; http://www.federa.org). A detailed description of the whole genome mRNA profiling has been previously published by Brandsma *et al.* and Hao *et al.*[1,2].

Asthma

Post mortem lung tissues from subjects with fatal asthma or subjects who died from nonpulmonary causes (controls) were retrieved from the Department of Pathology of São Paulo University (São Paulo, Brazil). Patient characteristics can be found in supplemental table 2. A detailed clinical and demographic description of this population has been previously published by Mauad *et al.*[3]. Diagnosis was confirmed by macro- and microscopic examination at autopsy and by an interview with the next of kin. Written informed consent was obtained with the next of kin. All experimental protocols within this study were approved by the institutional ethics committee Comissão de Ética para Análise de Projetos de Pesquisa - CAPPesq do Hospital das Clínicas, São Paulo University Medical School and were carried out in accordance with their guidelines. For this study we investigated the presence of TRAP activity in paraffin-embedded peripheral lung tissue samples of 10 asthma patients and 8 controls as described below.

Animal experiments

During the experiments, all animals were held under specific pathogen-free conditions in groups of 4-6 mice per cage in a temperature-controlled room with a 12h dark/light cycle and permanent access to food and water. The Groningen University Institutional Animal Care and Use Committee approved these experiments according to strict governmental and international guidelines on animal experimentation (DEC2857, DEC5318, and DEC6416AA-001).

Smoke-induced lung inflammation

To model COPD, we exposed five male A/JOlaHsd mice (Harlan, Horst The Netherlands, 8-10 weeks old) nose-only to mainstream cigarette smoke for 9 months in an experimental set-up as described before by us[4]. In short, mice were exposed daily to mainstream smoke from four 2R1 Reference Cigarettes (University of Kentucky, USA). This protocol was continued for 5 days/week for 9 months. Six control mice were sham-exposed to room air under similar conditions, following the same

duration of exposure as the smoke-exposed group. Mice were sacrificed after 9 months and lungs were collected, formalin-fixed and embedded in paraffin for histological analysis of TRAP activity.

Allergic lung inflammation

To model asthma, we exposed male and female BALB/c mice (Harlan, Horst The Netherlands, 8-10 weeks old) intranasally to whole body house dust mite (HDM) extract (*Dermatophagoides pteronyssinus*, Greer laboratories, Lenoir, USA) in 40 μl phosphate-buffered saline (PBS) according to a protocol we have described before[5]. In short, mice (n=8) were exposed to HDM extract under isoflurane anesthesia: one time to a high dose of HDM (100 μg) in the first week and 5 times to a low dose (10 μg) in the second week. Control animals (n=8) were exposed to 40 μl PBS according to this same schedule. Mice were sacrificed on day 24, three days after the last HDM exposure and the right lung was inflated with 0.5 ml 50% Tissue-Tek® O.C.T.™ compound (Sakura, Finetek Europe B.V., Zoeterwoude, The Netherlands) in PBS and formalin-fixed for histological analyses and TRAP activity. Other parameters of allergic lung inflammation of these animals are described in detail in our previous publication[5].

Enzyme histochemistry for TRAP activity

Presence of active TRAP was assessed using a histochemical method relying on conversion of chromogen Fast Red by active TRAP. In short, deparaffinated lung sections (3 μm) were rehydrated and incubated overnight in a buffer containing 0.1 M Tris at pH 7.4 supplemented with 30 mM calcium acetate, 23mM zinc acetate and 37mM zinc chloride, followed by pre-incubation in a 0.2 M acetate buffer at pH 5.0. TRAP-active cells were visualized by incubating sections for 0.5-4 hours at 37°C in a reaction solution containing 0.2 M acetate buffer with 0.5 mg/ml Naphtol AS-MX phosphate (Sigma-Aldrich, Zwijndrecht, The Netherlands) and 1.1 mg/ml fast red TR salt (Sigma-Aldrich) at pH 5.0. After hematoxylin counterstaining, sections were imbedded in VectaMount Permanent mounting medium (Vector Laboratories, Burlingame, CA). The number of positive

alveolar macrophages (based on morphology and tissue location) was counted manually with the aid of ImageScope software (Leica Biosystems, Son, The Netherlands) in human and murine lung tissue sections (on average a surface of 9 mm^2 was measured of each section) and corrected for the surface area of the corresponding lung tissue.

Cell culture of macrophages

Self-propagating murine alveolar-like macrophages (MPI macrophages, a kind gift from dr. G. Fejer, Plymouth University, Devon, UK) were cultured in RPMI 1640 medium (Gibco, Bleiswijk, The Netherlands) supplemented with 10% fetal bovine serum (FCS), gentamycin (10 μg/ml), and GM-CSF (20 ng/ml, Peprotech, Rocky Hill, USA) at 37°C under 5% CO_2 and humidified conditions as described by Fejer *et al.*[6]. MPI macrophages were plated at a density of 50,000 cells/well. The next day, cells were stimulated for 16h with a superoxide-generating system (0.2 mM Xanthine + 10mU/ml Xanthine oxidase (Sigma-Aldrich)) for 16 hrs to mimic oxidative stress, or for 24 hrs with the damage-associated molecular pattern ATP (1, 10 or 100 μg/ml, Sigma-Aldrich), RANKL (200 ng/ml, produced and provided by dr. R.H. Cool, University of Groningen, The Netherlands[7]), IL-4 (10 ng/ml, Peprotech), or M-CSF (10 ng/ml, Peprotech). Cells were harvested for mRNA isolation purposes.

RAW264.7 macrophages (American Type Culture Collection) were cultured in Dulbecco's modified Eagle's medium (Invitrogen, The Netherlands) supplemented with 10% FCS, 2mM L-glutamine, and Gentamycin (10 μg/ml) and cultured at 37°C, 5% CO_2, and humidified conditions. RAW264.7 macrophages were used in transwell and cell-tracking experiments, as further explained in "Inhibition of macrophage migration by AubipyOMe".

Quantitative Real-Time PCR

For TRAP mRNA expression, RNA was isolated using the Maxwell® LEV simply RNA Cells/Tissue kit

(Promega, Madison, WI). Final mRNA concentrations after isolation were determined using a Nanodrop ND-100 spectrophotometer (Nanodrop Technologies, Wilmington, DE). Subsequently, cDNA was prepared using 10 μl mRNA (20 μg/ml) with 5x RT-buffer, 10 mM dNTP's, 5 units Rnasin, random primers and 40 units M-MLV Rev transcriptase. The Eppendorf Thermocycler (Eppendorf, Hamburg, Germany) was used for the amplification of the cDNA. Transcription levels of TRAP were measured in 20 ng cDNA per sample in a quantitative real-time PCR (SensiMix™ SYBR kit, Bioline, Taunton, MA) and the ABI7900HT sequence detection system (Applied Biosystems, Foster City, USA). Primers used for RT-PCR were obtained from Sigma-Aldrich: TRAP forward: 5'-GCTGTCCTGGCTCAAAAAGC-3'; TRAP reverse: 5'-CACACCGTTCTCGTCCTGAA-3'; GAPDH forward: 5'-ACAGTCCATGCCATCACTGC-3'; GAPDH reverse: 5'-GATCCACGACGGACACATTG-3'. For each sample, the threshold cycles (Ct values) were calculated with the SDS 2.3 software program (Applied Biosystems) and mRNA expression was normalized against GAPDH. Experiments were repeated at least four times.

Precision-cut lung slices

Lungs of male C57BL/6 mice (20-30 gr) of in total six mice were used to make precision-cut lung slices. Mice were sacrificed under isoflurane anesthesia by exsanguination via the aorta abdominalis. Lungs were filled with low-melting temperature agarose (1,5% in 0.9% NaCl) (Sigma-Aldrich) and transferred directly into ice-cold University of Wisconsin organ preservation solution. Lung slices, diameter 5-mm and weight ±5 mg, were prepared with a Krumdieck tissue slicer (Alabama Research and Development, AL) using ice-cold Krebs-Henseleit Buffer [25 mM D-glucose (Merck, Darmstadt, Germany), 25 mM $NaHCO_3$ (Merck), 10 mM HEPES (MP Biomedicals, Aurora, OH), saturated with carbogen (95% O_2/5% CO_2) and adjusted to pH 7.4] as described by us before for liver slices[8].

After slicing, murine lung slices were transferred to 12-well plates with pre-warmed DMEM +

Glutamax medium (1.3 ml) [4.5g/L D-glucose and pyruvate (Gibco) supplemented with non-essential amino acid mixture (1:100), 100 U/ml penicillin, 100 μg/ml streptomycin, 45 μg/ml gentamycin, and 10% FCS]. Following 1h pre-incubation at 37°C in 95% O_2/5% CO_2 conditions and continuous shaking (90 rpm), slices were transferred into fresh medium and incubated in triplicate with the following stimulants: vehicle, RANKL (200 ng/ml), ATP (10 μg/ml), or Xanthine (0.2 mM) + Xanthine oxidase (10 mU/ml). After 24 hours of incubation, the three slices of each condition were pooled and directly snap frozen in liquid nitrogen and stored at -80°C. For the TRAP activity analysis, 400 μl acetate buffer was added to each sample. Slices were then homogenized for 45 sec using small glass pearls and a Mini-Beadbeater-24 (Biospec products, Bartlesville, UK), centrifuged (10 min, 13.2 rpm, 4°C), and used immediately for the TRAP activity assay.

TRAP activity assay on lysates of MPI macrophages and precision-cut lung slices

TRAP activity levels were determined in lung slice homogenates or MPI macrophage lysates by incubation at 37°C for 1 hour with an L-para-Nitrophenylphosphate (PNPP) solution [100 mM PNPP, 200 mM sodium citrate, 200 mM sodium chloride, 80 mM sodium tartrate, pH 4.5] at a 1:1 ratio. Absorption at 410 nm, with 490 nm as a reference value, was measured using a spectrophotometer. Each sample was measured in duplicate and stimulus outcome was calculated relative to the non-stimulated control absorption level.

Recombinant TRAP preparations and proteolytic digestion of TRAP

Recombinant unspecified human TRAP was purchased from R&D (Minneapolis, USA). Recombinant human TRAP5a and 5b were produced and purified according to a protocol based on several sources[9–11] using an ÄKTApurifier™ 10 FPLC system (GE Healthcare, Danderyd, Sweden) as previously described[12].

Identification of TRAP inhibitors

Initially, a small library of gold compounds was tested for TRAP inhibition using a TRAP activity assay with recombinant unspecified human TRAP (R&D). The Au(III) compounds [Au(terpy)Cl]Cl_2 (terpy = terpyridine, Auterpy), [Au_2(μ-O)$_2$(bipy)$_2$](PF_6)$_2$ (bipy = 2,2'-bipyridine, Auoxo) and [Au(bipyOMe)Cl_2][PF_6] (bipyOMe = 4,4'-dimethoxy-2,2'-bipyridine, AubipyOMe) were synthesized as previously described and their purity was confirmed by elemental analysis and showed to be >98 % [13–15]. The anti-rheumatic Au(I) compound sodium aurothiomalate and the reference Au(III) complex $NaAuCl_4$ were purchased from Sigma-Aldrich.

Inhibitor dilutions were prepared in acetate buffer from freshly prepared stock solutions (10 mM in DMSO). Recombinant unspecified TRAP (1.25 ng/ml, pH 4.5), TRAP5a (150ng/mL, pH 5) or TRAP5b (150 ng/mL, pH 5.8) were incubated at 37°C for 30 minutes with PNPP solution [10 mM PNPP, 200 mM sodium acetate, 300 mM potassium chloride) at the indicated pH at a 1:1 ratio and increasing concentrations of $NaAuCl_4$ (range 0-40 µM) or gold compounds (range 0-5.1 µM). To stop the reaction, 1M NaOH was added and absorption at 410 nm, with 490 nm as a reference value, was measured using a spectrophotometer.

Testing of inhibitors on cell and tissue lysates

Mouse alveolar macrophage lysates were obtained by resuspending 500.000 MPI macrophages in 300 µl acetate buffer. The cell suspension was sonicated, spun down (10 min, 13,200 rpm), and the supernatant was collected for TRAP activity assays. Human lung lysates from COPD patients were used to test the inhibitor on human TRAP present in lung tissue. Lysates from in total 18 COPD patients were pooled (see supplemental table 3 for patients characteristics). To obtain the tissue lysates, 10-20 mg frozen lung tissue was collected and 50 µl acetate buffer was added. Then, the tissue was homogenized manually using a small plunger. After spinning the samples down (13.200 rpm, 10 min, 4°C), all supernatants were pooled and stored at -80°C until use.

AubipyOMe was tested in the range 0-40 µM with MPI cell lysates or pooled lung tissue lysates from COPD patients using a TRAP activity assay. MPI lysates were diluted 1:1 and COPD tissue lysates 1:20 with acetate buffer and incubated with increasing concentrations inhibitor, that were prepared from freshly made stock solutions (10 mM in DMSO) and diluted with acetate buffer. Lysates were incubated at 37°C for 30 min in the presence of PNPP solution (1:1 ratio). 1M NaOH was added to stop the reaction and absorption was measured at 410 nm with 490 nm as a reference value.

IC50 calculations

The inhibitory effects of gold compounds were calculated as the ratio of absorbance between the treated and untreated wells. The IC50 values were calculated using nonlinear curve fitting with a variable slope in Graphpad Prism 6 (Graphpad Software, la Jolla, USA). An average IC50 of three independent experiments was calculated.

MTT assay

The effect of AubipyOMe and $NaAuCl_4$ on cell growth was assessed with a classical MTT assay. RAW264.7 macrophages were seeded in 96-well plates at a concentration of 10,000 cells/well and grown for 24h in complete medium. Compound dilutions were prepared in complete medium from a freshly prepared stock solution (10 mM in DMSO) and added to the wells (200 µl) to obtain a final concentration (0 to 100 µM). Cells were incubated for 72 h after which 3-(4,5-dimethylthiazol-2-yl)-2,5-diphenyltetrazolium bromide (MTT) was added to the cells at a final concentration of 0.50 mg/ml and incubated for 3h. Afterwards, the MTT-solution was removed and the violet formazan crystals dissolved in DMSO. The optical density of each well was quantified in quadruplicate at 540 nm, using a multi-well plate reader, and the percentage of surviving cells was calculated from the ratio of absorbance between treated and untreated cells. The IC50 values were calculated using nonlinear curve fitting with a variable slope in Graphpad Prism 6 (Graphpad Software) An average

IC50 of four independent experiments was calculated.

Transwell experiment to assess macrophage migration

Migration of RAW264.7 macrophages was assessed using a transwell-culturing system with inserts (Sigma-Aldrich) coated with osteopontin (10 μg/ml, R&D). Bovine collagen-coated inserts (Advanced Biometrix, Carlsbad, USA) were used as a matrix control. RAW264.7 macrophages, pre-stimulated with 200 ng/ml RANKL for 72h to induce TRAP activity[16], were seeded on 8 μm pore inserts coated with osteopontin (10 μg/ml, 250,000 cells/well) or collagen (10 μg/ml, 250,000 cells/well). Cells were cultured for 16h in the presence or absence of AubipyOMe (80 nM) in quadruplicate. The previously described TRAP inhibitor 5-PNA (100 μM) was used as selective positive control for TRAP inhibition[12,17]. The number of cells migrated to the lower well, including dead cells, was calculated relative to control cells and individual experiments were done at least six times.

Confocal imaging of macrophage migration

Briefly, RAW264.7 macrophages were plated on osteopontin-coated (10 μg/ml, R&D) Lab-tek chamber slides (Nunc, Hatfield, USA) at a density of 7500 cells/well. Cells were incubated with or without RANKL (200 ng/ml) for 48 hours to induce TRAP activity or not[16], followed by labeling with carboxyfluorescein diacetate succinimidyl ester (CFSE, Invitrogen, Life Technologies Europe BV, Bleiswijk, The Netherlands) to visualize cells for live cell imaging using a confocal microscope (Solamere Nipkow Confocal Live Cell Imaging system, Solamere Technology Group, Salt lake city, USA). A solution of 4μM CFSE in PBS was added to the cells for 20 min. After washing the cells with medium, new medium was applied with or without AubipyOMe (80 nM). Cell movement was tracked overnight in the presence of 1% zymosan solution (Sigma-Aldrich) to stimulate cell movement. Environmental conditions were kept at 37°C and 96% O_2/4% CO_2. Pictures were taken every 10 min and were transformed into movies with Image J and Imaris x64 (Bitplane, Zurich, Switzerland) software[18].

Statistical analysis

All data were assumed to have nonnormal distributions. Statistical differences between two groups were calculated using a Mann-Whitney *U* test. When comparing multiple groups, a Kruskal-Wallis with a Dunn's correction for multiple testing was performed. When studying correlations, a Spearman coefficient was calculated to test for significant relationships. $P<0.05$ was considered significant for all data. Statistical tests were done using Graphpad Prism 6 (Graphpad Software). Data are presented as Box and Whiskers plots with the whiskers representing the 2.5-97.5 percentile.

Supplementary figures

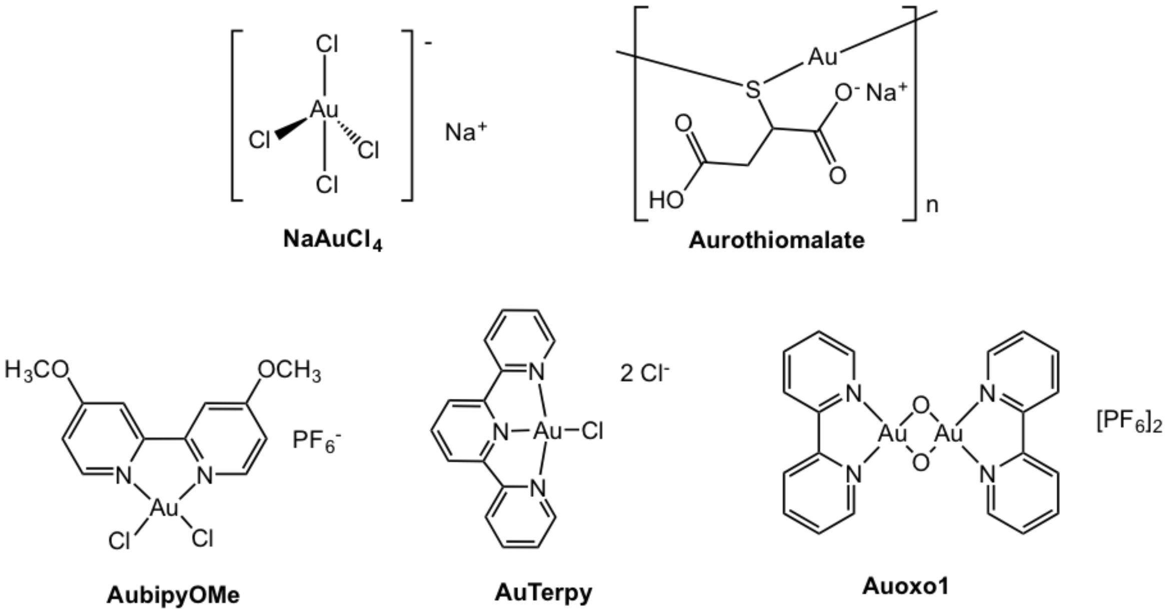

Figure S1. Chemical structures of the Au(III)/Au(I) compounds tested for their potential TRAP inhibitory capacities.

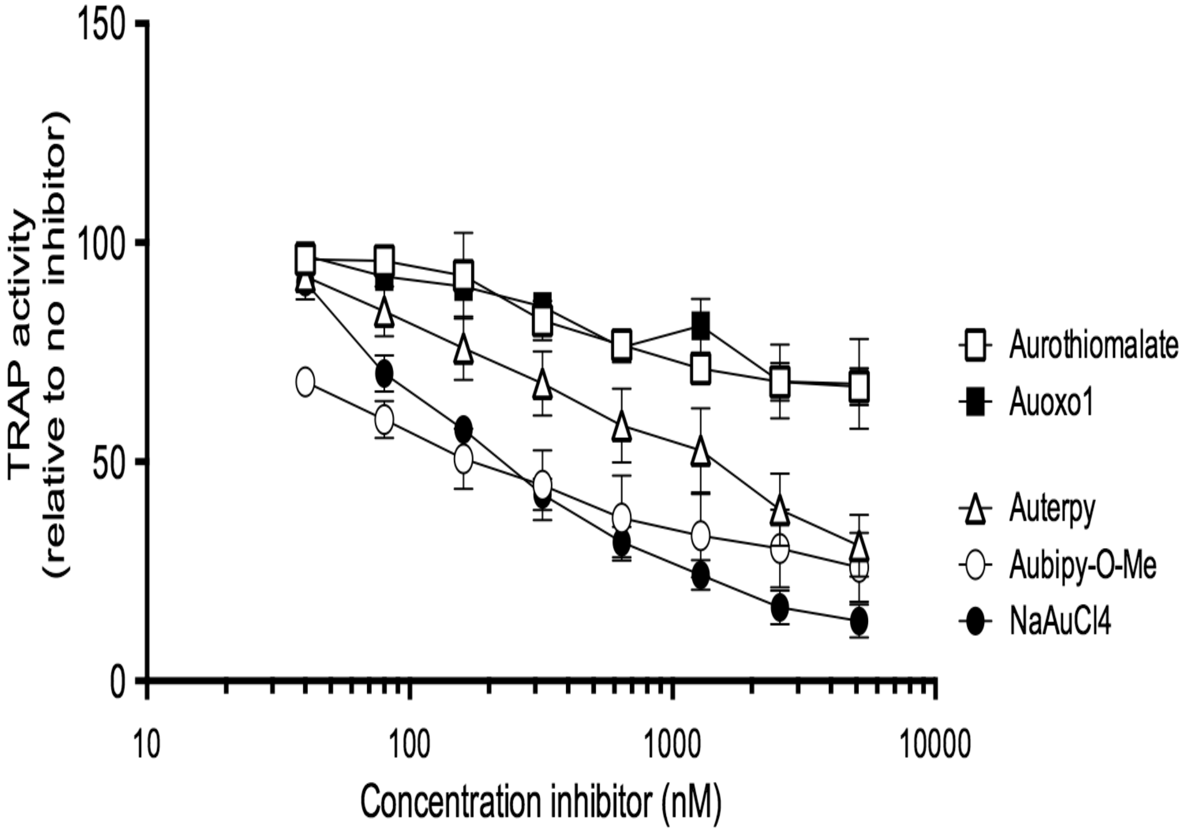

Figure S2. The inhibitory capacity of four different Au(III) coordination compounds on TRAP. Activity was assessed by using recombinant TRAP and incubating the enzyme for 30 min with each compound.

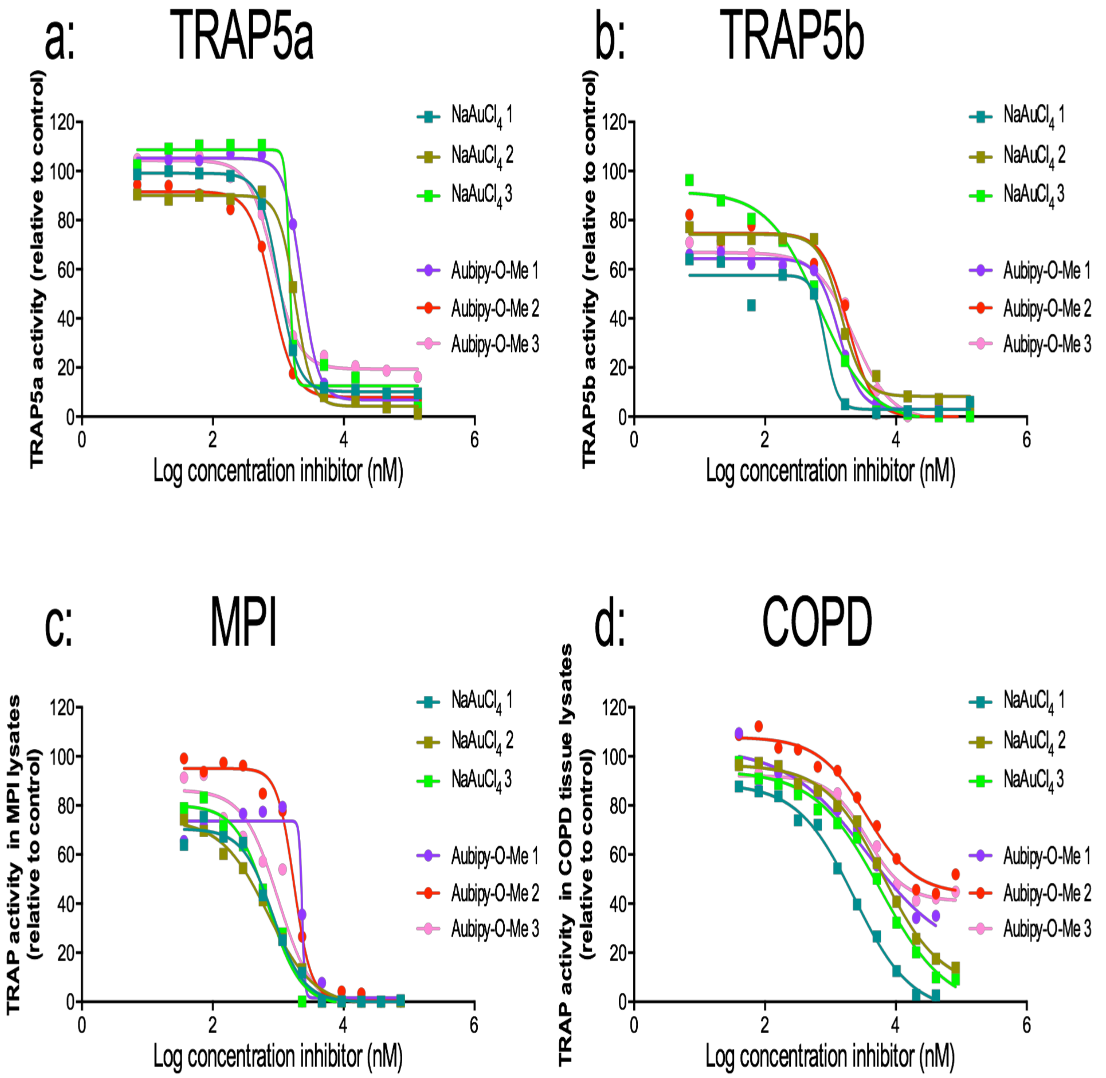

Figure S3. IC50 values for $NaAuCl_4$ and AubipyOMe in each individual experiment were calculated using nonlinear curve fitting with a variable slope after log transformation of the inhibitor concentrations. **a**: Fitted curves of $NaAuCl_4$ and AubipyOMe for TRAP5a inhibition. **b:** Fitted curves of $NaAuCl_4$ and AubipyOMe for TRAP5b inhibition. **c:** Fitted curves of $NaAuCl_4$ and AubipyOMe for TRAP inhibition in MPI cell lysate. **d:** Fitted curves of $NaAuCl_4$ and AubipyOMe for TRAP inhibition in COPD tissue lysate.

Tables

Table S1. Patient characteristics of patients with and without fatal asthma (median + range is displayed).

	**Control (n=8)**	**Fatal asthma (n=10)**
**Sex, male/female**	5/3	5/5
**Age, years**	49.5 (37-58)	35 (20-66)
**Smoking history:**		
**Nonsmokers**	8	5
**Exsmokers**	0	0
**Corticosteroid use:**		
**Oral**	0	2
**Inhaled**	0	0
**Cause of death:**		
**Asthma**	0	10
**Cardiovascular disease**	7	0
**Acute pancreatitis**	1	0

Table S2. Patient characteristics of COPD patients from whom lung tissue was used for the preparation of lung tissue lysates (median + range is displayed).

	**COPD (n=18)**
**Sex, male/female**	12/6
**Age, years**	66 (35-80)
**Smoking status**	18 ex-smoker
**FEV_1 % pred.**	62 (36-77)
**GOLD stage**	GOLD II = 15<br>GOLD III = 3

Table S3. Patient characteristics of control and COPD patients used to assess TRAP mRNA expression (median + range is displayed).

	**Control (n=270)**	**COPD (n=311)**
**Sex, male/female**	145/125	182/129
**Age, years**	61 (37-80)	63 (33-84)
**Smoking status**	197 ex-smoking<br>73 current smoking	222 ex-smoking<br>89 current smoking
**FEV_1 % pred.**	98% (80-136)	57 (11-83)
**FEV_1/FVC**	76 (70-91)	53 (16-70)
**GOLD stage**	-	GOLD I = 1, GOLD II = 216<br>GOLD III = 27, GOLD IV = 56 Undetermined = 11

Movies 1-4

Live cell tracking of RAW264.7 macrophages on osteopontin-coated wells revealed that macrophage migration was higher in the presence of RANKL (200ng/ml) as compared to control and AubipyOMe (80 nM) inhibited this mobility. **Movie 1:** Live cell tracking of untreated control RAW264.7 macrophages; **Movie 2:** Live cell tracking of RANKL-treated (200ng/ml) RAW264.7 macrophages; **Movie 3:** Live cell tracking of RANKL-treated (200ng/ml) RAW264.7 macrophages in the presence of TRAP inhibitor AubipyOMe (80ng/ml); **Movie 4:** Live cell tracking of untreated control RAW264.7 macrophages in the presence of TRAP inhibitor AubipyOMe (80ng/ml).

References

1. Brandsma, C. A. et al. A large lung gene expression study identifying fibulin-5 as a novel player in tissue repair in COPD. *Thorax* **70**, 21-32 (2015).

2. Hao, K. et al. Lung eQTLs to help reveal the molecular underpinnings of asthma. *PLoS Genet* **8**, e1003029 (2012).

3. Mauad, T. et al. Characterization of autopsy-proven fatal asthma patients in São Paulo, Brazil. *Rev Panam Salud Publica* **23**, 418-423 (2008).

4. van der Strate, B. W. A. et al. Cigarette smoke-induced emphysema: A role for the B cell? *Am J Respir Crit Care Med* **173**, 751-758 (2006).

5. Draijer, C., Robbe, P., Boorsma, C. E., Hylkema, M. N. & Melgert, B. N. Characterization of Macrophage Phenotypes in Three Murine Models of House-Dust-Mite-Induced Asthma. *Mediators Inflamm* **2013**, 1-10 (2013).

6. Fejer, G. et al. Nontransformed, GM-CSF-dependent macrophage lines are a unique model to study tissue macrophage functions. *Proc Natl Acad Sci U S A* **110**, E2191-8 (2013).

7. Wang, Y. et al. Novel RANKL DE-loop mutants antagonize RANK-mediated osteoclastogenesis. *FEBS J* (2017).

8. Olinga, P. et al. Rat liver slices as a tool to study LPS-induced inflammatory response in the liver. *J Hepatol* **35**, 187-194 (2001).

9. Ek-Rylander, B. et al. Comparative studies of rat recombinant purple acid phosphatase and bone tartrate-resistant acid phosphatase. *Biochem J* **321**, 305-311 (1997).

10. Zenger, S., Ek-Rylander, B. & Andersson, G. Biogenesis of tartrate-resistant acid phosphatase isoforms 5a and 5b in stably transfected MDA-MB-231 breast cancer epithelial cells. *Biochim Biophys Acta* **1803**, 598-607 (2010).

11. Igarashi, Y., Lee, M. Y. & Matsuzaki, S. Heparin column analysis of serum type 5 tartrate-resistant acid phosphatase isoforms. *J Chromatogr B Biomed Sci Appl* **757**, 269-276 (2001).

12. Krumpel, M. et al. The small chemical enzyme inhibitor 5-phenylnicotinic acid/CD13 inhibits cell migration and invasion of tartrate-resistant acid phosphatase/ACP5-overexpressing MDA-MB-231 breast cancer cells. *Exp Cell Res* **339**, 154-162 (2015).

13. Hollis, L. S. & Lippard, S. J. Aqueous chemistry of (2,2′,2″-terpyridine)gold(III). Preparation and structures of chloro(2,2′,2″-terpyridine)gold dichloride trihydrate ([Au(terpy)Cl]Cl2.3H2O) and the mixed valence gold(I)-gold(III) salt bis[chloro(2,2′,2″-terpyridine)gold] tris(dichloroaurate) tetrachloroaurate ([Au(terpy)Cl]2[AuCl2]3[AuCl4]). *J Am Chem Soc* **105**, 4293-4299 (1983).

14. Casini, A., Cinellu, M. A. & Minghetti…, G. Structural and solution chemistry, antiproliferative effects, and DNA and protein binding properties of a series of dinuclear gold (III) compounds with bipyridyl ligands. *… medicinal chemistry* (2006).

15. Casini, A. et al. Synthesis, characterisation and biological properties of gold(III) compounds with modified bipyridine and bipyridylamine ligands. *Dalton Trans* **39**, 2239-2245 (2010).
16. Karlström, E., Ek-Rylander, B., Wendel, M. & Andersson, G. RANKL induces components of the extrinsic coagulation pathway in osteoclasts. *Biochem Biophys Res Commun* **394**, 593-599 (2010).
17. Feder, D. et al. Identification of purple acid phosphatase inhibitors by fragment-based screening: promising new leads for osteoporosis therapeutics. *Chem Biol Drug Des* **80**, 665-674 (2012).
18. Schneider, C. A., Rasband, W. S. & Eliceiri, K. W. NIH Image to ImageJ: 25 years of image analysis. *Nat Methods* **9**, 671-675 (2012).
